# Microplastic concentrations, size distribution, and polymer types in the surface waters of a northern European lake

**DOI:** 10.1002/wer.1229

**Published:** 2019-09-12

**Authors:** Emilia Uurasjärvi, Samuel Hartikainen, Outi Setälä, Maiju Lehtiniemi, Arto Koistinen

**Affiliations:** ^1^ SIB Labs University of Eastern Finland Kuopio Finland; ^2^ Department of Environmental and Biological Sciences University of Eastern Finland Kuopio Finland; ^3^ Marine Research Centre Finnish Environment Institute Helsinki Finland

**Keywords:** fourier‐transform infrared, freshwater, Lake, microplastics, plastic pollution

## Abstract

We examined microplastic concentrations, size distributions, and polymer types in surface waters of a northern European dimictic lake. Two sampling methods, a pump sieving water onto filters with different pore sizes (20, 100, and 300 µm) and a common manta trawl (333 µm), were utilized to sample surface water from 12 sites at the vicinity of potential sources for microplastic emissions. The number and polymer types of microplastics in the samples were determined with optical microscopy and μFTIR spectroscopy. The average concentrations were 0.27 ± 0.18 (mean ± *SD*) microplastics/m^3^ in manta trawled samples and 1.8 ± 2.3 (>300 μm), 12 ± 17 (100–300 μm) and 155 ± 73 (20–100 μm) microplastics/m^3^ in pump filtered samples. The majority (64%) of the identified microplastics (*n* = 168) were fibers, and the rest were fragments. Materials were identified as polymers commonly used in consumer products, such as polyethylene, polypropylene, and polyethylene terephthalate. Microplastic concentrations were high near the discharge pipe of a wastewater treatment plant, harbors, and snow dumping site.

**Practitioner points:**

- Samples were taken with a manta trawl (333 μm) and a pump filtration system (300/100/20 μm)
- With pump filtration, small 20–300 μm particles were more common than >300 μm particles
- The average concentration of manta trawled samples was 0.27 ± 0.18 (mean ± *SD*) microplastics/m^3^
- FTIR analysis revealed PE, PP, PET, and PAN to be the most common polymers

## Graphical abstract

Microplastics in lake surface waters were sampled with a manta trawl and analyzed fith FTIR.

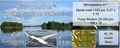

## Introduction

the increasing production of plastic and the accumulation of plastic waste in marine environments has raised concern on the impacts of plastics on environment (Barnes, Galgani, Thompson, & Barlaz, 2009). Because of deficiencies in the waste recycling and processing, the amount of plastic accumulating in the environment has been enormous during this “plastic era” we are living in (Jambeck et al., 2015). Microplastics (MPs) are defined as plastic debris with a particle size of 1–1,000 μm (Hartmann et al., 2019), or 1–5,000 μm (GESAMP, 2015). MPs end up to the environment by the fragmentation of plastic debris, the wear and tear of plastic items, and the leakage of intentionally manufactured small plastic particles MPs are insoluble to water and consist of polymer(s) and additives, such as plasticizers, colorants, and flame retardants (Hahladakis, Velis, Weber, Iacovidou, & Purnell, 2018).

Studies on MP abundance in the marine environment demonstrate their worldwide presence (Auta, Emenike, & Fauziah, 2017). The first MP studies were carried out in marine environment, but recently also freshwater bodies have gained increasing attention. Building up knowledge on (micro)plastics in freshwaters is vital, because shallow freshwater bodies are vulnerable to pollution, they provide drinking water, and may act as routes from land‐based MP sources to sea (Wagner et al., 2014). To date, MPs have been studied in surface waters, for example, in Laurentian Great Lakes of the United States (Eriksen et al., 2013), Lake Winnipeg in Canada (Anderson et al., 2017), Lake Hovsgol in Mongolia (Free et al., 2014), and Taihu Lake in China (Su et al., 2016). In Europe, microplastics of lake surface waters have been studied in Swiss (Faure, Demars, Wieser, Kunz, & Alencastro, 2015) and Italian lakes (Fischer, Paglialonga, Czech, & Tamminga, 2016), and in Hungarian natural and excavated lakes (Bordos et al., 2019).

Central European lakes are typically small and lie in populated areas. Contradictory, lakes of Scandinavia and Finland in northern Europe are larger, lie in less populated areas, and differ in biotic and abiotic factors. For example, boreal lakes are dimictic (the water mixes completely from surface to bottom twice a year with temperature changes) and have permanent ice cover during the winter. These unique conditions may affect MPs. Therefore, it is important to collect and publish microplastic data from northern lakes, because it is currently not available. To our best knowledge, this is the first published MP survey in Nordic lake environments and one of the few lake MP studies, which utilize spectroscopic methods for polymer analysis.

Our aim is to examine concentrations, size distributions, polymer types, and potential sources of MPs in Lake Kallavesi, located in Eastern Finland. In addition, we sampled with two methods providing complementary information and evaluated the suitability of them for lake water sampling. In the case of Lake Kallavesi, several potential sources for MP emissions are compactly located on the meandering lakeshore, which makes it an optimal sampling site for tracking MP sources.

## Materials and Methods

### Sampling

Lake Kallavesi is the tenth largest lake in Finland with a surface area of 478.1 km^2^, mean depth of 9.7 m, and maximum depth of 75 m (Miettinen & Lindholm, 2018). It contains over 1,900 islands, is fed from a drainage basin of 16,270 km^2^, and is covered by ice on average 170 days/year. Lake Kallavesi provides drinking water to the city of Kuopio (population: 118,000), surrounded by the lake.

**Figure 1:**
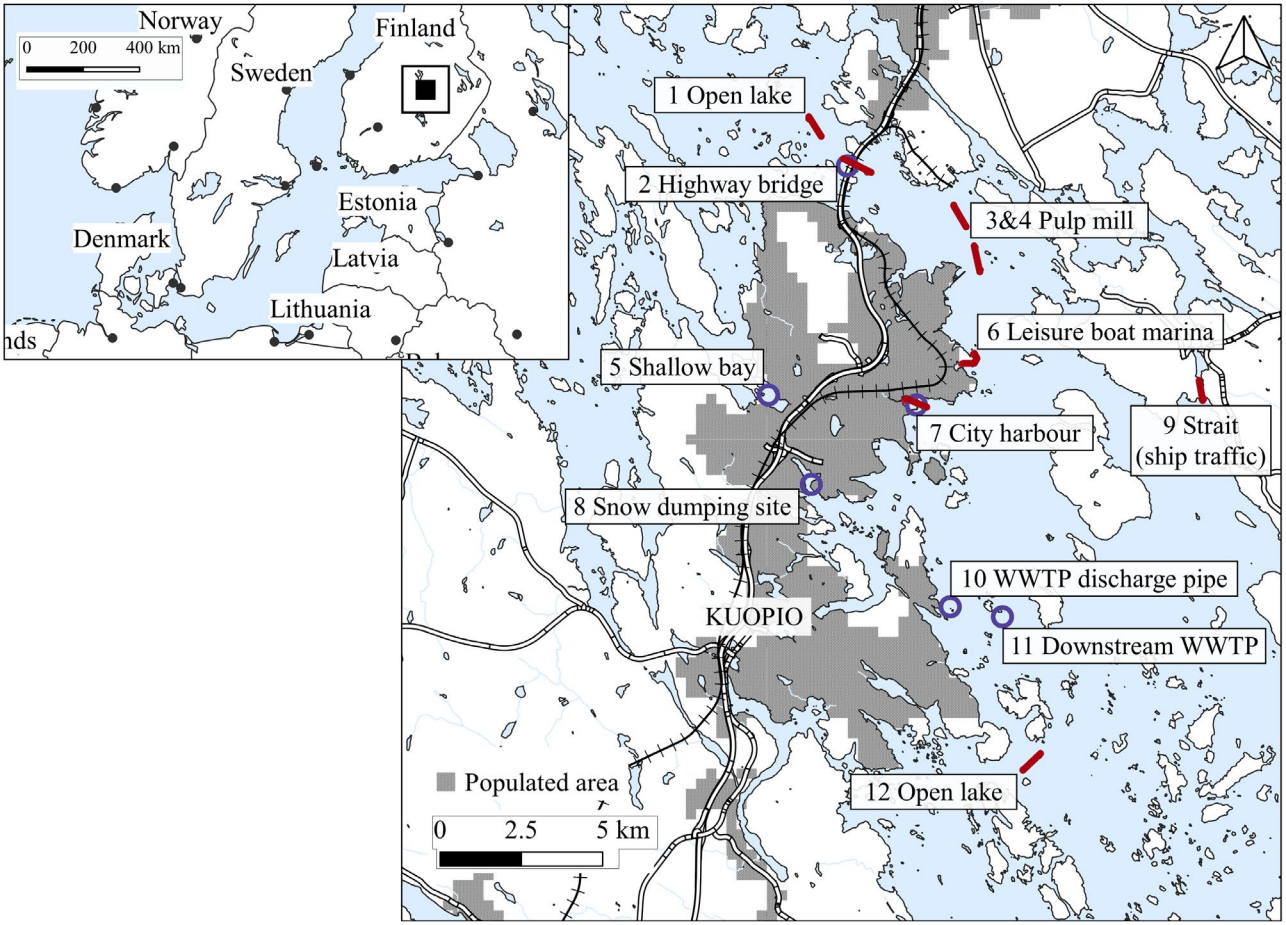
Sampling sites. WWTP = wastewater treatment plant, red line = manta trawl, blue circle = pump filtration.

We collected surface water samples from Lake Kallavesi with two methods, a manta trawl (333 μm) in autumn 2016, and a pump with a filtration device in spring 2017. Samples were taken before or after the dimictic mixing times, and the sampling depth was approximately 0–16 cm. Sampling sites (Figure 1), which represent potential local sources for MPs, were compared with samples from open lake area (sites 1, 12). Sampling sites were near a highway bridge (2), close to the discharge pipe of a pulp mill (3–4), in a shallow bay (5), close to harbors (6–7), in a snow dumping site (8), in a narrow strait with inhabitance and ship traffic (9) and close to a wastewater treatment plant's (WWTP) discharge pipe (10–11). Manta trawl samples were taken from sites 1, 2, 3, 4, 6, 7, 9, and 12, whereas pump filtration was used at sites 2, 5, 7, 8, 10, and 11.

Because the manta trawl is a commonly used sampling method for collecting MPs from surface waters (Li, Liu, & Paul Chen, 2018), we chose it to sample larger particles. We used a suitcase manta trawl (designed and manufactured by Marcus Eriksen, 5 Gyres Institute), which has a rectangular opening of 16 cm (height) × 61 cm (width) and the net mesh size of 333 μm. The manta trawl was towed on the side of the vessel for approximately 10 min at a speed of 2.5 knots. After the tows, net contents were rinsed with tap water to 5‐L buckets (food‐grade polypropylene), which were closed immediately with polypropylene lids. The water volumes sampled with the manta trawl were calculated based on the opening area of the trawl and a flow rate meter. The average sampled volume was 58 ± 14 m^3^, and one tow was taken per site.

The pump filtration was conducted in addition to manta trawling, because it was expected to be able to provide data about smaller particles. Moreover, our study lake has several narrow and shallow bays, where pump filtration was more practical than trawling. In the pump filtration device, a petrol‐driven pump was used to suck surface water through a custom‐made cascade‐like filtering tube made from PVC (Talvitie et al., 2015). The tube has three screw joints, which hold three filters vertically. The filters were cut from plankton net (nylon), and their pore sizes were 300, 100, and 20 μm, giving particle size fractions of >300, 100–300, and 20–100 μm. Water was pumped to the tube via PVC hose placed on the water surface. When the filters with smaller pores were clogged, they were removed from the tube and the filtration was continued with the larger one(s). The average sampled volumes in all sites were 6.2 ± 1.8 L with 20 μm, 83 ± 37 L with 100 μm, and 468 ± 75 L with 300 μm filter. Samples were taken from the lake in duplicates, and the results are presented as an average of the two samples. After sampling, filters were removed from the filtering tube and stored immediately in closed Petri dishes (Greiner CELLSTAR, PS, 100 × 20 mm).

### Sample pretreatment

All containers and equipment were rinsed thoroughly before use with distilled and filtered ultrapure water. Pump filtration samples required no further pretreatments before material characterization, because the filters contained only minor amount of solids. Manta samples, rinsed to buckets, were digested with NaOH (VWR International) and sodium dodecyl sulfate (SDS, VWR International). Digestion was done in the same buckets to prevent contamination. First, NaOH was added while stirring until pH reached 12. Then, SDS (1 g/L) was added. Buckets were incubated in 50°C in a water bath for 4 hr with stirring. Thereafter, they were incubated overnight without stirring at the same temperature. The digested sample solution was neutralized with HCl (VWR International) and filtered to Whatman 114 (25 μm) cellulose filters. The filters were stored in closed Petri dishes (Greiner CELLSTAR, PS, 100 × 20 mm) in a fridge for further analysis.

### Microscopy and FTIR analysis

All filters with samples were visually inspected with a stereo microscope (Zeiss Stemi 508; 6.3–50× magnification; Axiocam ERc 5s camera; Carl Zeiss Microscopy GmbH, Jena, Germany). From every sample, all particles having plastic‐like appearance were manually selected from the filters with micro tweezers and placed on ZnSe transmission windows. Particles were imaged with the stereo microscope's camera. Particles were considered as plastic‐like if they met the following criteria (Noren, 2007): (a) the structure was elastic and durable—no breaking when handling with tweezers, (b) the color was uniform, and non‐natural or particle was colorless/transparent, (c) the shape was equally thick fibrous or smooth fragment. It has to be considered that the actual cutoff size was somewhat higher than 20 μm because of the optical microscopy and manual selection step. Despite, results are presented by filter pore sizes for clarity.

Fourier‐transform infrared (FTIR) spectra were measured from each plastic‐like particle. Particles on ZnSe windows were measured with an FTIR microscope (PerkinElmer Spectrum Spotlight; PerkinElmer, Waltham, MA, USA) in point mode with an MCT detector. Measurements were done in transmission, using aperture 25 × 25 µm, spectral resolution 4 cm^−1^, number of scans 16, and spectral range 4,000–700 cm^−1^.

Spectra were analyzed with Thermo OMNIC 9 software (Thermo Fisher Scientific, Waltham, MA, USA). The spectra measured were compared to a commercial polymer library (Hummel Polymer Sample Library, Thermo Fisher Scientific) and a custom‐made synthetic fiber library (Talvitie, Mikola, Setälä, Heinonen, & Koistinen, 2017). The correlation search was done from the entire spectral range, and the recognition limit was set to 70%. However, spectra were also individually interpreted to ensure the recognition, because some of the MPs were weathered or dirty. Only the particles, which were confirmed to be plastic, were calculated to the results. Microscope images and FTIR spectra of particles are available in the Supporting information.

### Contamination controls

Contamination from the laboratory environment during the microscope and FTIR analysis was estimated with three control samples (C1–C3). Controls were clean and dry Whatman 114 filters on open Petri dishes. They were exposed to laboratory air for 3 hr, 1 hr and 75 min, in different days during the sample analysis. The controls were examined for the presence of MPs with the stereo microscope and analyzed with μFTIR similarly as the actual samples.

## Results

### Concentrations and sizes

#### Manta samples

**Figure 2:**
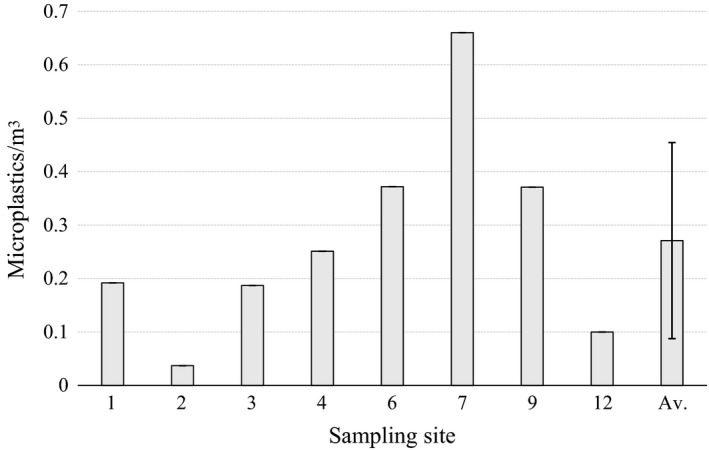
Microplastics (MP) concentrations sampled with the manta trawl in different sampling sites. Site numbers are shown and explained in Figure 1. Av. = Average MP concentration, error bar = *SD*.

The average MP concentration of manta trawl samples was 0.27 ± 0.18 MPs/m^3^ (mean ± *SD*) (Figure 2). The highest concentration (0.66 MPs/m^3^) was observed at site 7, near the city harbor, and the lowest (0.037 MPs/m^3^) at site 2, under the highway bridge. Samples from sites 1, 3, 4, and 12 (open lake and pulp mill) had also lower concentrations than the average. On the other hand, samples from the sites 6 and 9 (leisure boat marina and strait with ship traffic) contained more MPs than the average.

#### Pump filtration samples

**Figure 3:**
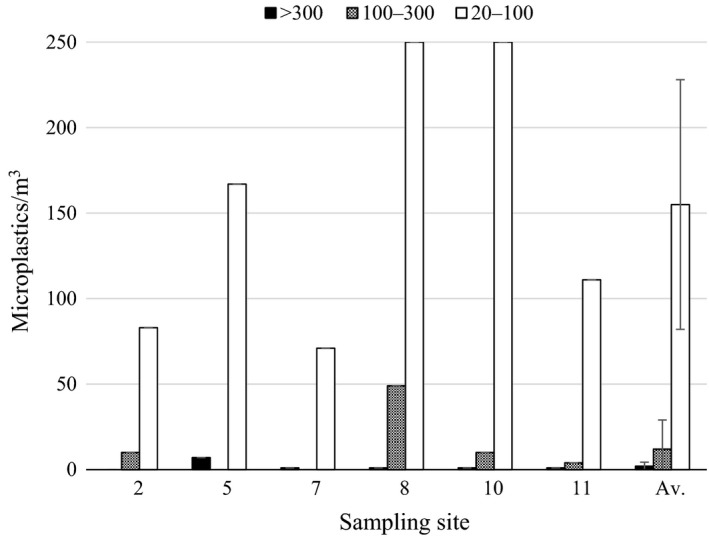
Microplastics (MP) concentrations of three size fractions sampled with pump filtration. Sampling site numbers are shown and explained in Figure 1. Av. = Average MP concentrations, error bars = *SD*.

The average concentrations of pump filtration samples over all sampling sites were 1.8 ± 2.3 (>300 μm), 12 ± 17 (100–300 μm) and 155 ± 73 (20–100 μm) MPs/m^3^ (mean ± *SD*) (Figure 3). The variations between sampling sites were quite high. Site 8, by the snow dumping site, had the highest MP concentration and site 7, City harbor, the lowest. Site 10, WWTP discharge pipe area, had also high concentration (Figure 3).

Generally, the amount of MPs/m^3^ increased with a decreasing filter pore size. Pump filtration with 300 μm filter pore size provided higher average MP concentrations (1.8 ± 2.3 MPs/m^3^) compared to the MP concentrations in samples collected with the >333 μm manta trawl (0.27 ± 0.18 MPs/m^3^). However, manta and pump filtration datasets cannot be strictly compared to each other, because the sampling methods are different and have been utilized in different places during different seasons.

### Morphology and chemical composition

**Figure 4:**
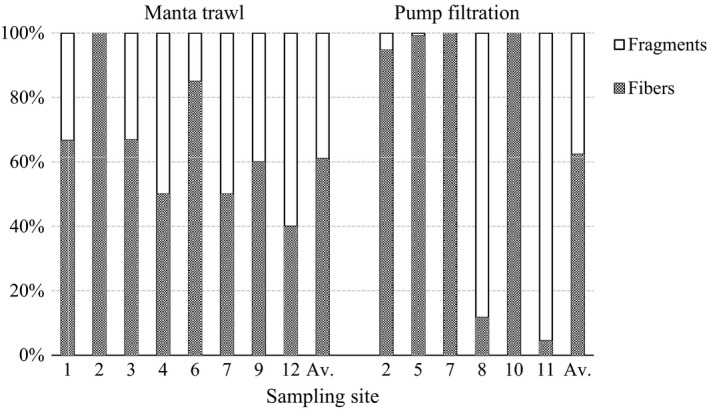
Distributions of concentrations of fibers and fragments in different sampling sites. In the pump filtration bars, the concentrations of different size fractions are summed. Site numbers are shown and explained in Figure 1. Av. = Average distribution of concentrations.

All visually detected plastic‐like particles in the samples were handpicked (495 particles), and every one of them was measured with μFTIR, which confirmed 34% of them as MPs. A total of 138 MPs were found from manta trawl samples and 30 from pump filtration samples, resulting in 168 MPs overall. Of these, 64% (107) were synthetic fibers and 36% (61) fragments. Figure 4 presents the distributions of concentrations of fibers and fragments for both sampling methods.

**Figure 5:**
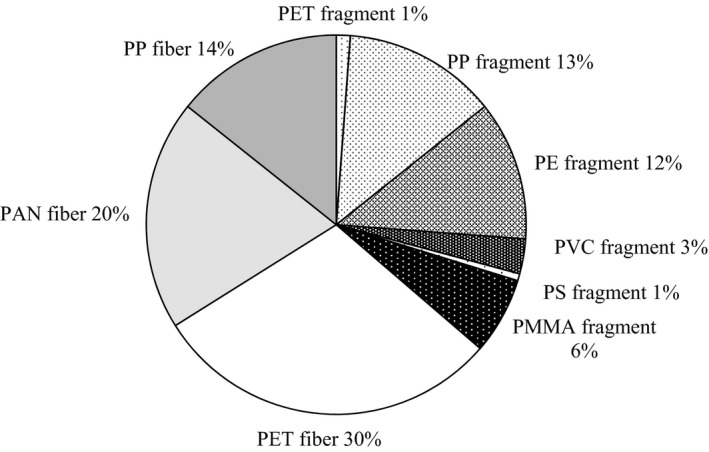
Polymer types and morphologies of microplastics.

Fragments were irregularly shaped three‐dimensional particles with various colors, such as white, blue, green, and red. FTIR analysis revealed them to be polyethylene (PE), polypropylene (PP), polymethyl methacrylate (PMMA), polyvinyl chloride (PVC), polyethylene terephthalate (PET), and polystyrene (PS) (Figure 5). PP and PE were the most common polymer types in fragments. Plastic fibers were of several colors; white, blue, red, brown, black, and green, and they consisted of PP, PET, and acryl (polyacrylonitrile, PAN).

In addition to MPs, high amount of cellulose fibers were found. Those were not taken into account, because the recognition of different cellulose materials was quite uncertain. Cellulose fibers can originate from manufactured materials, such as textiles and paper, and natural materials such as plants. Resolving the cellulose origin with FTIR would have required more insightful approach than exploited in this study, because samples contained much biological material from the lake (Comnea‐Stancu, Wieland, Ramer, Schwaighofer, & Lendl, 2017). Moreover, as the contamination control indicated (see below), that risk for contamination was present especially regarding cellulose fibers.

### Contamination controls

Control C1 contained nine fibers, which all were identified to be cellulose materials (cotton and viscose). Control C2 had only one fiber, which was cellulose. The last control C3 contained two fibers. First of them could not be identified, but the second one was composed of cellulose. The controls indicated that fibers float in the laboratory air and easily contaminate open filters. Based on this analysis, the amount of fiber contamination increases with exposure time. The open examination time of sample filters was approximately one hour or less, depending on the amount of plastic‐like particles. It can be estimated that the fiber contamination will be small or negligible when the amount of selected particles is low. In this study, cellulose fibers were not taken into account in the analysis. Thus, we estimated the air‐borne contamination to be negligible for our results in the laboratory analysis step. Other sources of contamination, such as reagents and containers, were also possible but not examined.

## Discussion

### Comparison to other studies

Both surface trawling and pumping method have been applied in marine and freshwater environments (Fischer et al., 2016; Hidalgo‐Ruz, Gutow, Thompson, & Thiel, 2012). The average MP concentration in samples collected with the manta trawl from Lake Kallavesi (0.27 ± 0.18 MPs/m^3^) corresponds with the earlier study from the Baltic Sea, where in average 0.2 ± 0.2 MPs/m^3^ were detected (Setälä, Magnusson, Lehtiniemi, & Norén, 2016). Our pump filtration results showed that MP concentration increases with decreasing particle size, also shown by Railo, Talvitie, Setälä, Koistinen, and Lehtiniemi (2018) for the Baltic Sea. However, microplastics in the smallest two size fractions (20–100 and 100–300 μm) were found to be more abundant in Lake Kallavesi samples than what was found from the coastal waters of the Gulf of Finland (Railo et al., 2018).

Only a few studies have examined concentrations and polymer types of MPs in lake waters. For example in Italy, surface water samples from Lake Chiusi contained 2.68–3.36 and Lake Bolsena 0.82–4.42 MPs/m^3^ >300 μm particles (Fischer et al., 2016). MP concentrations in these small lakes in densely populated Italy were higher than in large Lake Kallavesi, which lies in a less populated area. Relatively high concentrations were also found in Hungarian surface waters of natural and excavated lakes (3.52–32.05 MPs/m^3^, 100 μm–2 mm particles) (Bordos et al., 2019). This result is higher than our mean pump filtration concentration for >100 μm particles.

However, when concentrations of different size fractions are compared, particle number per volume may not be the best unit for reporting. Because particles degrade continuously, it is not a conserved quantity (Simon, Alst, & Vollertsen, 2018). Although a filter with smaller pores collects higher number of particles, the overall plastic mass, which is a conserved quantity, also matters. Because particle size seems to affect MP uptake of aquatic animals (Lehtiniemi et al., 2018), particle sizes, numbers and masses per sample would all be necessary to report in future studies. In this study, the analysis method was not suitable for measuring or estimating masses.

### Variation between sampling sites

Manta trawl and pump filtration datasets are not comparable to each other, because the sampling methods are different and have been utilized in different places during different seasons. Therefore, we discuss only the variations found within each dataset.

In the manta trawling, the highest concentrations were found near the urban areas compared to open lake. Samples from harbors had high concentrations of MPs, which were mostly fibers (PP, PAN, PET), but also plastic fragments (PE, PP, PMMA, PS). The variety of detected polymer materials and particle morphologies was high, which indicates that they originate from multiple sources. The highway bridge sampling site showed a low amount of MPs possibly because of the strong currents below the bridge area. However, roads, road markings and especially car tires are estimated to be significant sources of MP emissions to environment (Jan Kole, Löhr, Belleghem, & Ragas, 2017). The knowledge of average tire wear particle size is limited, but they might be too small (<20 μm) to sample and analyze with the method used in this study, or so dense that they sink to the bottom. Additionally, tires contain 22%–40% carbon black, which hinders the FTIR characterization. Near the pulp mill, concentrations were close to average and MP types were fibers (PET, PAN) and fragments (PE, PP, PMMA). Fiber/fragment concentration ratio was also near the average in these sites.

In the pump filtered samples, snow dumping site had the highest MP concentrations. Snow, which contains plastic and other litter, is collected from city streets to the dumping site, located on the lakeshore. In the city of Kuopio, snow covers ground on average 160–175 days/year, and it has to be removed from the compact city center. MPs found from the lake near that site in spring after the melting of the snow were mainly PVC, PE, and PET fragments, which may originate from large plastic litter thrown to the streets. The second highest pump filtered concentration was found near the WWTP discharge pipe. In that site, all the identified MPs were acrylic and polyester fibers, which probably indicates that they result from wastewater loaded by washing of synthetic textiles (Sillanpää & Sainio, 2017), although 98%–99% of MPs are removed during the treatment process (Murphy, Ewins, Carbonnier, & Quinn, 2016; Talvitie et al., 2017). In comparison, 88% of synthetic particles found near snow dumping site were fragments. In downstream WWTP discharge pipe, concentration was lower than the average and found MPs were mainly fragments. Highway bridge and city harbor had also lower concentrations. Samples from shallow urban bay contained slightly higher concentration of MPs than average, mostly fibers.

The city harbor had the highest concentration in manta samples, but the lowest in pump filtration. Moreover, the ratio of concentrations of fibers and fragments was different between the sample sets. Manta samples were taken with larger mesh from a long transect in autumn, whereas pump filtration was done with multiple mesh sizes from one point in spring. In addition, filtered volumes were smaller in pump filtration. Some or many of these factors affect the results prominently. Possibly, MP concentrations are higher in autumn than in spring, or the harbor contains larger particles more than smaller. However, resolving the reason behind this would require more sampling with both methods concurrently.

### Polymer types and morphology

In all samples, fibers were more common than fragments. Fibers consisted of PET, PAN, and PP polymers, which are used, for example, in textiles and industrial processes (GESAMP, 2015). Fiber lengths varied largely, and some of them were shiny and uniform, whereas others had breaks and small branches. Therefore, MP fibers were difficult or impossible to distinguish from natural fibers with optical microscopy and only the μFTIR step revealed the chemical composition of the particles.

All of the fragment polymers (PP, PE, PMMA, PVC, PS, and PET) are commonly used in consumer products, such as packages, bottles, coatings, films, and construction materials (GESAMP, 2015). Symmetrical or spherical MPs were not observed, but because primary MPs, such as manufactured microbeads, can be also irregularly shaped (Fendall & Sewell, 2009), categorization of MPs to primary or secondary was impossible. Most of the found MP fragments were PE and PP polymers, which float on the water surface. Denser polymers, which should sink toward the bottom, were less common in surface water samples.

Most of the previous MP studies of lake surface waters have not utilized spectroscopic methods and do not provide information about chemical composition. However, in Hungarian freshwaters PE, PP, PS, and polyester were the most common polymer types (Bordos et al., 2019). Additionally, in Lake Superior, the United States, the most common polymers were PE, PET, PVC, and PP, analyzed with FTIR (Hendrickson, Minor, & Schreiner, 2018). Nevertheless, MPs in lake sediments have more widely been characterized spectroscopically. In Canadian Lake Ontario, PE, PS, polyurethane (PU), PP, and PVC were the most common polymer types in sediment samples (Anderson et al., 2017). Comparably, sediments of Italian Lake Garda contained primarily low‐density polymers such as PS, PE, and PP and smaller amount of polyamide (PA) and PVC (Imhof, Ivleva, Schmid, Niessner, & Laforsch, 2013). PE, PP, and PET were also abundant in our study, but we found only a couple of PS particles.

PU and PA were not found in our study. PA might have degraded in sample treatment process, left unidentified with FTIR, or it is not very common in our sampling area. Manta samples were treated with NaOH, which has been reported to degrade PA (Roch & Brinker, 2017). Besides, PA is often difficult to recognize with FTIR if biofilm covers it. Biological material contains amino acids linked by amide bonds, thus it has similar features in FTIR spectrum than PA, which may cause misidentifications.

### Method validity and accuracy

The manta trawl and a similarly functioning pump filtration system than utilized here have previously been compared and discussed in detail for marine sampling (Setälä et al., 2016). In freshwater sampling, both sampling methods had pros and cons. The manta trawl is suitable for filtering larger water volumes from longer area, but the pump filtration is suitable for collecting also smaller particles and many size fractions simultaneously. The cascade‐like structure of filtration tube helps to prevent the clogging of smallest pored filters, and largest (pre)filter can be analyzed too. Comparing the two sampling methods, manta trawling is suitable for open water areas, whereas pump filtration is more practical in small water bodies and coastal sampling. Thus, the choice of sampling method depends on the geography of sampling site and target particle size(s).

Considering contamination and method validation, the pump filtration tube, which is a closed system, is less sensitive to contamination and can be validated with procedural blanks (Talvitie et al., 2015). It is also possible to do recovery tests for the tube with spiked samples, and we have previously studied that the MP recovery of a similar pump filtration tube for tap water samples was 91% (unpublished results). However, the hose and tube are made of plastic (PVC), which may cause contamination. In this study, five PVC particles were found from three sampling sites. Snow dumping site contained three PVC particles, others only one. Because the amount of PVC particles was quite low, it is likely that sampling system does not release many larger >50 μm particles during one sampling event. Especially when the analysis method can detect very small particles, it is still important to wash and rinse the sampling system well before use and do always blanks to ensure that the tube and hose do not contaminate samples.

The exact estimation of contamination is impossible when using the manta trawl, because it is not possible to take a blank sample with it. Additionally, it is not possible to do recovery tests for manta sampling. On the other hand, the pump filtration data might be more vulnerable to random variation, because the filtered water volumes and detected MP numbers were rather low, compared to the manta samples. Because of clogging, the average pumped water volume in 20–100 μm samples was only 6.2 ± 1.8 L (mean ± *SD*), which might not be representative for m^3^. Despite this, results of 20–100 μm fractions were reported per m^3^ to ease the comparison with other fractions. In the future, the filter combinations could be adjusted for filtering larger volumes by changing the smallest pore size to, for example, 50 μm, or using 100/50/20 μm filter set for collecting small particles. We used plankton net filters made from nylon, but stainless steel filters have also been used for freshwater sampling (Bordos et al., 2019).

We collected each particle suspected to be of plastic origin and analyzed the polymer type of every single particle. The analysis showed that only about 34% of visually selected particles were actually MPs. This result emphasizes the need to examine microliter by spectroscopic or other suitable analytical method, as pointed out previously (Rocha‐Santos & Duarte, 2015). Recent studies report fully automatic μFTIR spectroscopic analysis with a focal plane array detector (Primpke, Wirth, Lorenz, & Gerdts, 2018). That procedure could be, when applicable, the most efficient and accurate method for MP determination, if MPs can be satisfyingly isolated and recovered from the sample matrix.

In our analysis method, the risk of false negative was remarkably high, because potential plastic particles were selected by visual inspection. Manta samples contained large amounts of algae, insects, and other biological material and particles were selected from the partially digested matrix remains, consisting mainly of chitin. More efficient digestion method, which dissolves also chitin, could ease this problem (Löder et al., 2017). Additionally, the digestion step made the manta method more prone to contamination than the pump method. All of the collected particles were spectroscopically identified to be plastic or other materials with sufficient accuracy. The risk of false positive was therefore very low without counting the possible contamination. However, contamination from equipment and reagents was not analyzed, and the controls likely underestimated the amount of contamination coming from the whole sampling and analysis process.

## Conclusions

Microplastic concentrations and polymer types in dimictic northern European lakes have not been studied before. This study fulfills the gap by providing data about concentrations and polymer types in surface waters of a Finnish lake. Currently, researchers analyze MPs with a wide variety of methods. For freshwater sampling, both manta trawling and pump filtration are suitable, depending on what kind of areas and particle sizes are on the focus. However, the pump and filtration method is more versatile, because pore sizes of filter sets can be easily adjusted and the system allows method validation with blanks and spiked samples. Micro‐spectroscopic methods are appropriate for MP analysis, because they can provide information on both chemical composition and particle size. In the future, more comprehensive monitoring would be necessary for wider knowledge of the spatial and temporal patterns of MP abundance.

## Conflicts of Interest

The authors declare no conflicts of interest. Data are available on request from the authors.

## References

[wer1229-bib-0001] Anderson, P. J. , Warrack, S. , Langen, V. , Challis, J. K. , Hanson, M. L. , & Rennie, M. D. (2017). Microplastic contamination in Lake Winnipeg, Canada. Environmental Pollution, 225, 223–231. 10.1016/j.envpol.2017.02.072 28376390

[wer1229-bib-0002] Auta, H. S. , Emenike, C. U. , & Fauziah, S. H. (2017). Distribution and importance of microplastics in the marine environment: A review of the sources, fate, effects, and potential solutions. Environment International, 102, 165–176. 10.1016/j.envint.2017.02.013 28284818

[wer1229-bib-0003] Barnes, D. K. A. , Galgani, F. , Thompson, R. C. , & Barlaz, M. (2009). Accumulation and fragmentation of plastic debris in global environments. Philosophical Transactions of the Royal Society B: Biological Sciences, 364, 1985–1998. 10.1098/rstb.2008.0205 PMC2873009 19528051

[wer1229-bib-0004] Bordos, G. , Urbanyi, B. , Micsinai, A. , Balazs, K. , Palotai, Z. , Szabo, I. , … Szoboszlay, S. (2019). Identification of microplastics in fish ponds and natural freshwater environments of the Carpathian basin, Europe. Chemosphere, 216, 110–116. 10.1016/j.chemosphere.2018.10.110 30359912

[wer1229-bib-0005] Comnea‐Stancu, I. R. , Wieland, K. , Ramer, G. , Schwaighofer, A. , & Lendl, B. (2017). On the identification of rayon/viscose as a major fraction of microplastics in the marine environment: Discrimination between natural and manmade cellulosic fibers using fourier transform infrared spectroscopy. Applied Spectroscopy, 71, 939–950. 10.1177/0003702816660725 27650982 PMC5418941

[wer1229-bib-0006] Eriksen, M. , Mason, S. , Wilson, S. , Box, C. , Zellers, A. , Edwards, W. , … Amato, S. (2013). Microplastic pollution in the surface waters of the Laurentian Great Lakes. Marine Pollution Bulletin, 77, 177–182. 10.1016/j.marpolbul.2013.10.007 24449922

[wer1229-bib-0007] Faure, F. , Demars, C. , Wieser, O. , Kunz, M. , & de Alencastro, L. F. (2015). Plastic pollution in Swiss surface waters: Nature and concentrations, interaction with pollutants. Environmental Chemistry, 12, 582–591. 10.1071/EN14218

[wer1229-bib-0008] Fendall, L. S. , & Sewell, M. A. (2009). Contributing to marine pollution by washing your face: Microplastics in facial cleansers. Marine Pollution Bulletin, 58, 1225–1228. 10.1016/j.marpolbul.2009.04.025 19481226

[wer1229-bib-0009] Fischer, E. K. , Paglialonga, L. , Czech, E. , & Tamminga, M. (2016). Microplastic pollution in lakes and lake shoreline sediments – A case study on Lake Bolsena and Lake Chiusi (central Italy). Environmental Pollution, 213, 648–657. 10.1016/j.envpol.2016.03.012 27104923

[wer1229-bib-0010] Free, C. M. , Jensen, O. P. , Mason, S. A. , Eriksen, M. , Williamson, N. J. , & Boldgiv, B. (2014). High‐levels of microplastic pollution in a large, remote, mountain lake. Marine Pollution Bulletin, 85, 156–163. 10.1016/j.marpolbul.2014.06.001 24973278

[wer1229-bib-0011] GESAMP (2015). Sources, fate and effects of microplastics in the marine environment: A global assessment. Kershaw P. J. (Ed.) (IMO/FAO/UNESCO‐IOC/UNIDO/WMO/IAEA/UN/UNEP/UNDP Joint Group of Experts on the Scientific Aspects of Marine Environment Protection). Reports and Studies GESAMP, No. 90, 96 p.

[wer1229-bib-0012] Hahladakis, J. N. , Velis, C. A. , Weber, R. , Iacovidou, E. , & Purnell, P. (2018). An overview of chemical additives present in plastics: Migration, release, fate and environmental impact during their use, disposal and recycling. Journal of Hazardous Materials, 344, 179–199. 10.1016/j.jhazmat.2017.10.014 29035713

[wer1229-bib-0013] Hartmann, N. B. , Huffer, T. , Thompson, R. C. , Hassellov, M. , Verschoor, A. , Daugaard, A. E. , … Wagner, M. (2019). Are we speaking the same language? Recommendations for a definition and categorization framework for plastic debris. Environmental Science and Technology, 53, 1039–1047. 10.1021/acs.est.8b05297 30608663

[wer1229-bib-0014] Hendrickson, E. , Minor, E. C. , & Schreiner, K. (2018). Microplastic abundance and composition in western lake superior as determined via microscopy, Pyr‐GC/MS, and FTIR. Environmental Science and Technology, 52, 1787–1796. 10.1021/acs.est.7b05829 29345465

[wer1229-bib-0015] Hidalgo‐Ruz, V. , Gutow, L. , Thompson, R. C. , & Thiel, M. (2012). Microplastics in the marine environment: A review of the methods used for identification and quantification. Environmental Science and Technology, 46, 3060–3075. 10.1021/es2031505 22321064

[wer1229-bib-0016] Imhof, H. K. , Ivleva, N. P. , Schmid, J. , Niessner, R. , & Laforsch, C. (2013). Contamination of beach sediments of a subalpine lake with microplastic particles. Current Biology, 23, R867–R868. 10.1016/j.cub.2013.09.001 24112978

[wer1229-bib-0017] Jambeck, J. R. , Geyer, R. , Wilcox, C. , Siegler, T. R. , Perryman, M. , Andrady, A. , … Law, K. L. (2015). Plastic waste inputs from land into the ocean. Science, 347, 768–771. 10.1126/science.1260352 25678662

[wer1229-bib-0018] Jan Kole, P. , Löhr, A. J. , Van Belleghem, F. G. A. J. , & Ragas, A. M. J. (2017). Wear and tear of tyres: A stealthy source of microplastics in the environment. International Journal of Environmental Research and Public Health, 14, 1265 10.3390/ijerph14101265 PMC5664766 29053641

[wer1229-bib-0019] Lehtiniemi, M. , Hartikainen, S. , Näkki, P. , Engström‐Öst, J. , Koistinen, A. , & Setälä, O. (2018). Size matters more than shape: Ingestion of primary and secondary microplastics by small predators. Food Webs, 17, e00097 10.1016/j.fooweb.2018.e00097

[wer1229-bib-0020] Li, J. , Liu, H. , & Paul Chen, J. (2018). Microplastics in freshwater systems: A review on occurrence, environmental effects, and methods for microplastics detection. Water Research, 137, 362–374. 10.1016/j.watres.2017.12.056 29580559

[wer1229-bib-0021] Löder, M. G. J. , Imhof, H. K. , Ladehoff, M. , Löschel, L. A. , Lorenz, C. , Mintenig, S. , … Gerdts, G. (2017). Enzymatic purification of microplastics in environmental samples. Environmental Science and Technology, 51, 14283–14292. 10.1021/acs.est.7b03055 29110472

[wer1229-bib-0022] Miettinen, T. , & Lindholm, M. (2018). Kallavesi – Järviwiki. Retrieved from http://www.jarviwiki.fi/wiki/Kallavesi_(yhd.)

[wer1229-bib-0023] Murphy, F. , Ewins, C. , Carbonnier, F. , & Quinn, B. (2016). Wastewater treatment works (WwTW) as a source of microplastics in the aquatic environment. Environmental Science and Technology, 50, 5800–5808. 10.1021/acs.est.5b05416 27191224

[wer1229-bib-0024] Noren, F. (2007). Small plastic particles in Coastal Swedish waters. KIMO Report.

[wer1229-bib-0025] Primpke, S. , Wirth, M. , Lorenz, C. , & Gerdts, G. (2018). Reference database design for the automated analysis of microplastic samples based on Fourier transform infrared (FTIR) spectroscopy. Analytical and Bioanalytical Chemistry, 410, 5131–5141. 10.1007/s00216-018-1156-x 29978249 PMC6113679

[wer1229-bib-0026] Railo, S. , Talvitie, J. , Setälä, O. , Koistinen, A. , & Lehtiniemi, M. (2018). Application of an enzyme digestion method reveals microlitter in Mytilus trossulus at a wastewater discharge area. Marine Pollution Bulletin, 130, 206–214. 10.1016/j.marpolbul.2018.03.022 29866549

[wer1229-bib-0027] Roch, S. , & Brinker, A. (2017). Rapid and efficient method for the detection of microplastic in the gastrointestinal tract of fishes. Environmental Science and Technology, 51, 4522–4530. 10.1021/acs.est.7b00364 28358493

[wer1229-bib-0028] Rocha‐Santos, T. , & Duarte, A. C. (2015). A critical overview of the analytical approaches to the occurrence, the fate and the behavior of microplastics in the environment. TrAC Trends in Analytical Chemistry, 65, 47–53. 10.1016/j.trac.2014.10.011

[wer1229-bib-0029] Setälä, O. , Magnusson, K. , Lehtiniemi, M. , & Norén, F. (2016). Distribution and abundance of surface water microlitter in the Baltic Sea: A comparison of two sampling methods. Marine Pollution Bulletin, 110, 177–183. 10.1016/j.marpolbul.2016.06.065 27339742

[wer1229-bib-0030] Sillanpää, M. , & Sainio, P. (2017). Release of polyester and cotton fibers from textiles in machine washings. Environmental Science and Pollution Research, 24, 19313–19321. 10.1007/s11356-017-9621-1 28669092

[wer1229-bib-0031] Simon, M. , van Alst, N. , & Vollertsen, J. (2018). Quantification of microplastic mass and removal rates at wastewater treatment plants applying Focal Plane Array (FPA)‐based Fourier Transform Infrared (FT‐IR) imaging. Water Research, 142, 1–9. 10.1016/j.watres.2018.05.019 29804032

[wer1229-bib-0032] Su, L. , Xue, Y. , Li, L. , Yang, D. , Kolandhasamy, P. , Li, D. , & Shi, H. (2016). Microplastics in Taihu Lake, China. Environmental Pollution, 216, 711–719. 10.1016/j.envpol.2016.06.036 27381875

[wer1229-bib-0033] Talvitie, J. , Heinonen, M. , Pääkkönen, J. P. , Vahtera, E. , Mikola, A. , Setälä, O. , & Vahala, R. (2015). Do wastewater treatment plants act as a potential point source of microplastics? Preliminary study in the coastal Gulf of Finland, Baltic Sea. Water Science and Technology, 72, 1495–1504. 10.2166/wst.2015.360 26524440

[wer1229-bib-0034] Talvitie, J. , Mikola, A. , Setälä, O. , Heinonen, M. , & Koistinen, A. (2017). How well is microlitter purified from wastewater? – A detailed study on the stepwise removal of microlitter in a tertiary level wastewater treatment plant. Water Research, 109, 164–172. 10.1016/j.watres.2016.11.046 27883921

[wer1229-bib-0035] Wagner, M. , Scherer, C. , Alvarez‐Muñoz, D. , Brennholt, N. , Bourrain, X. , Buchinger, S. , … Reifferscheid, G. (2014). Microplastics in freshwater ecosystems: What we know and what we need to know. Environmental Sciences Europe, 26, 1–9. 10.1186/s12302-014-0012-7 PMC5566174 28936382

