# Supplementary material for: Microplastic concentrations, size distribution, and polymer types in the surface waters of a northern European lake
Source: Water Environ Res. 2019 Sep 12;92(1):149–56. doi: 10.1002/wer.1229 (PMC6973047; doi:10.1002/wer.1229)
Supplement: Supplementary file 1 [file WER-92-149-s001.docx]

Supporting Information

Manuscript title: Microplastic concentrations, size distribution, and polymer types in the surface waters of a northern European lake

Authors: Emilia Uurasjärvi, Samuel Hartikainen, Outi Setälä, Maiju Lehtiniemi, Arto Koistinen

**Stereo microscope images and FTIR spectra of microplastics**

Particles were picked under a stereo microscope, placed on ZnSe transmission windows for μFTIR measurement, and imaged with the microscope’s camera. ZnSe windows were divided to nine sections with a permanent marker. One or two particles were placed on each section, and particles were numbered. This helped to locate particles for measurement in the FTIR microscope. Every particle has an ID, which is linked to a microscope image and an FTIR spectrum. Examples of microscope images and FTIR spectral analysis are shown in Figures S1 – S10.


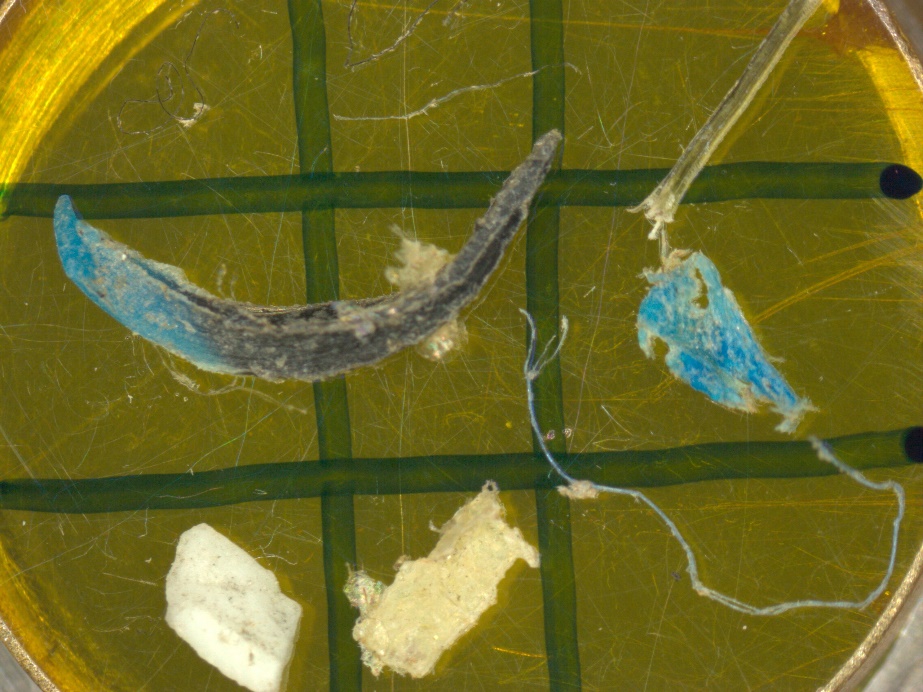


Figure S1. Particles from manta trawling in site 7, the city harbor (10X magnification). The green fragment on the top right corner is cellulose (plant material), the blue and white large fragments are PE or PP and the blue fiber is PP. Small black fibers on the top are PET fibers.


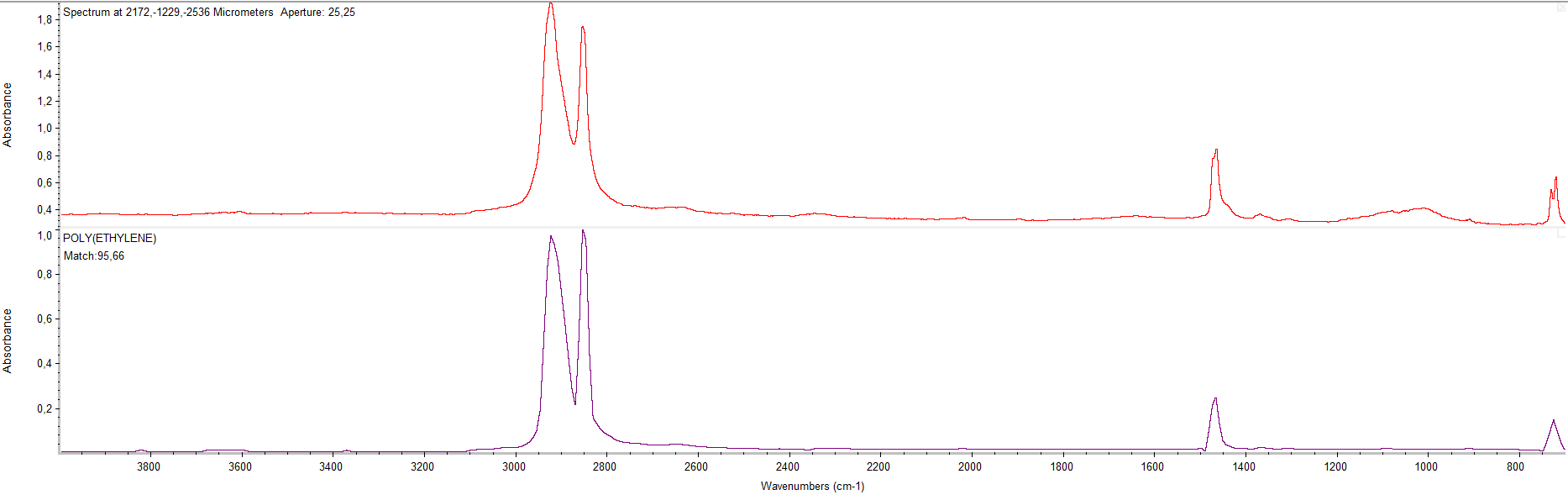


Figure S2. FTIR spectrum and spectral library search result of the large blue/black particle in Figure 1.


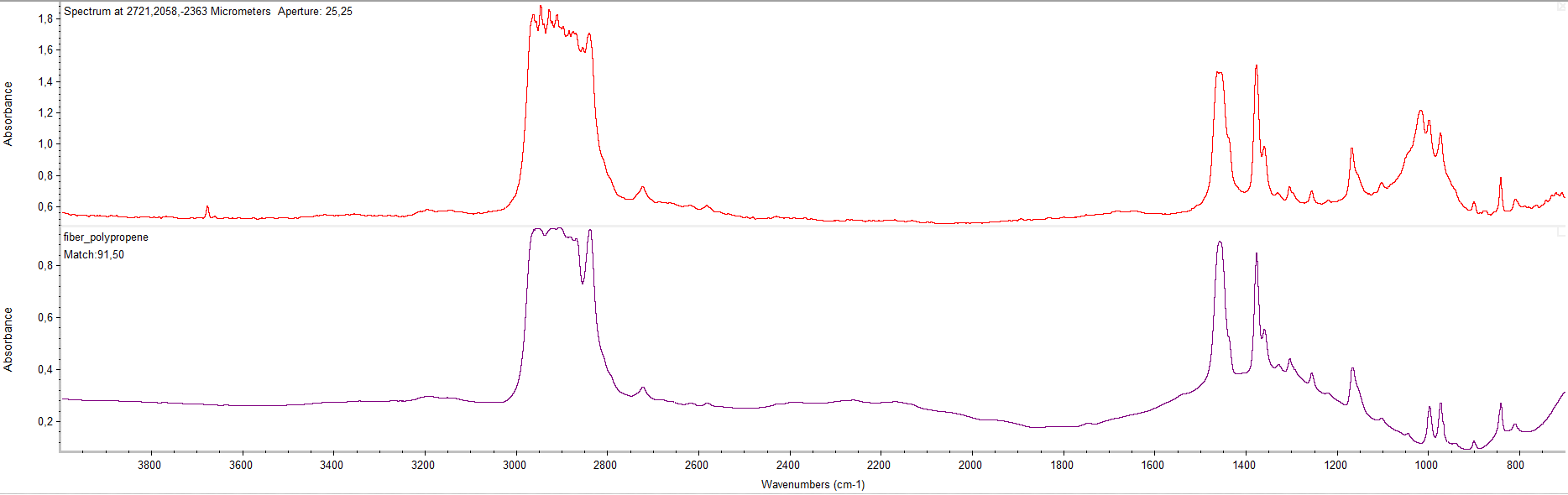


Figure S3. FTIR spectrum and spectral library search result of the white particle in the lower left corner in Figure 1.


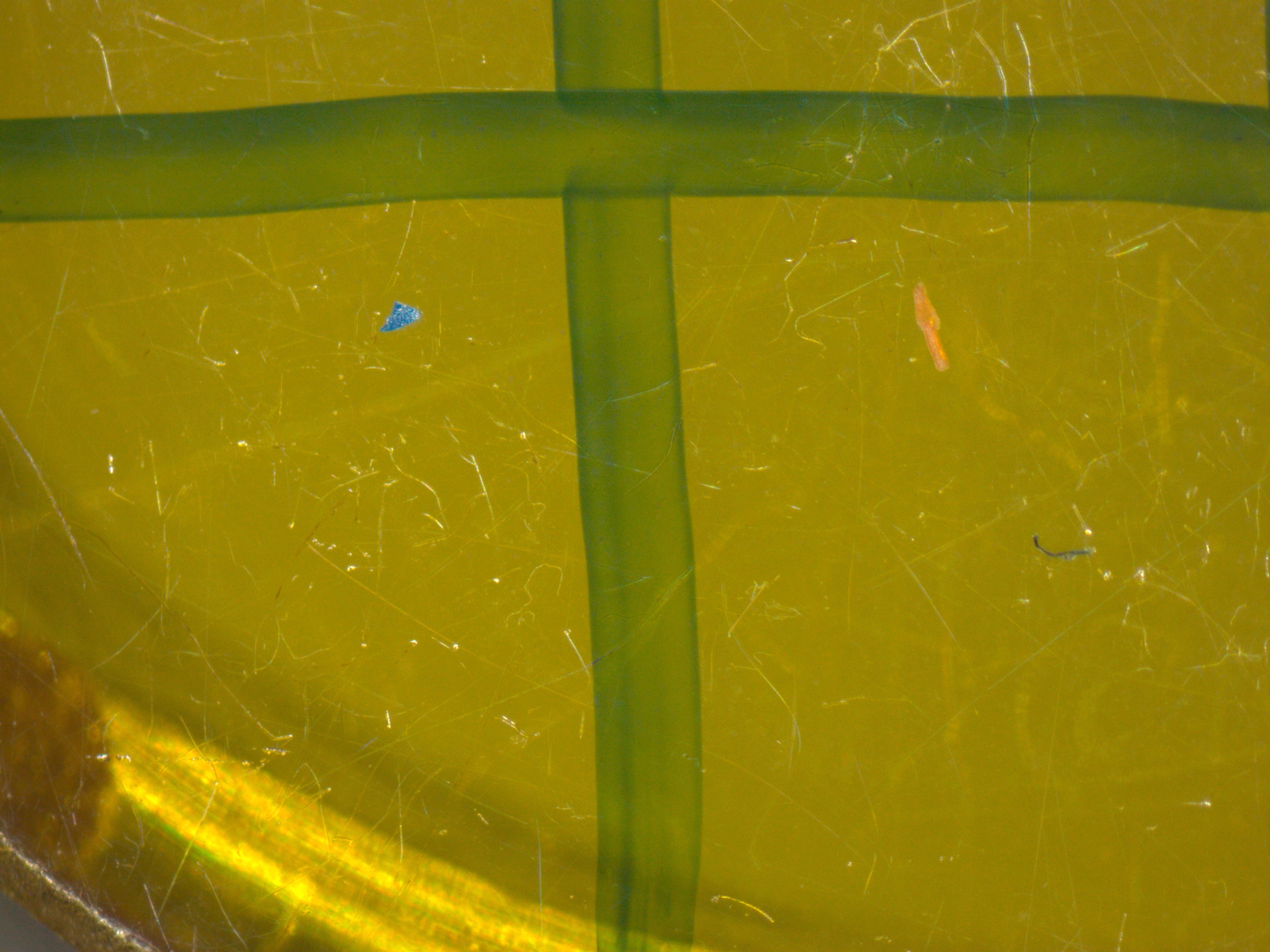


Figure S4. Particles sampled with pump filtration and 20 μm filter from site 11, downstream WWTP. The blue fragment is PET, red fragment is PP and small blue fiber is cellulose material.


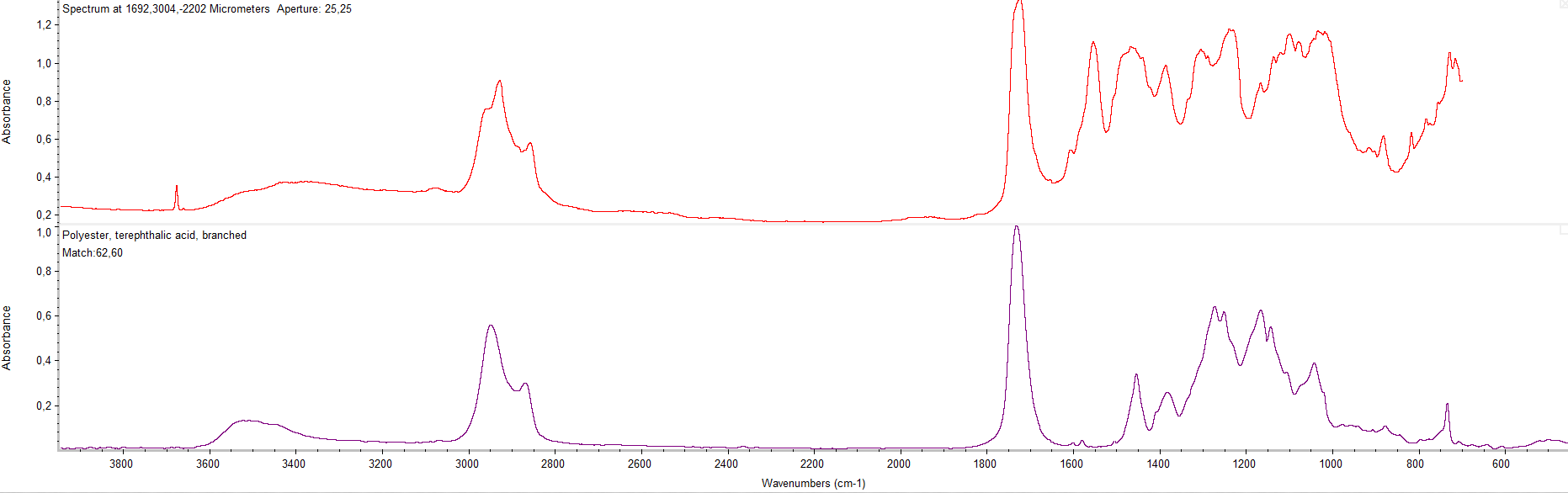


Figure S5. FTIR spectrum and spectral library search results of the small flue fragment in Figure 4. Correlation (match) is not very good because of for example the intense amide II peak in 1556 cm^-1^, caused by biological remains in the sample.


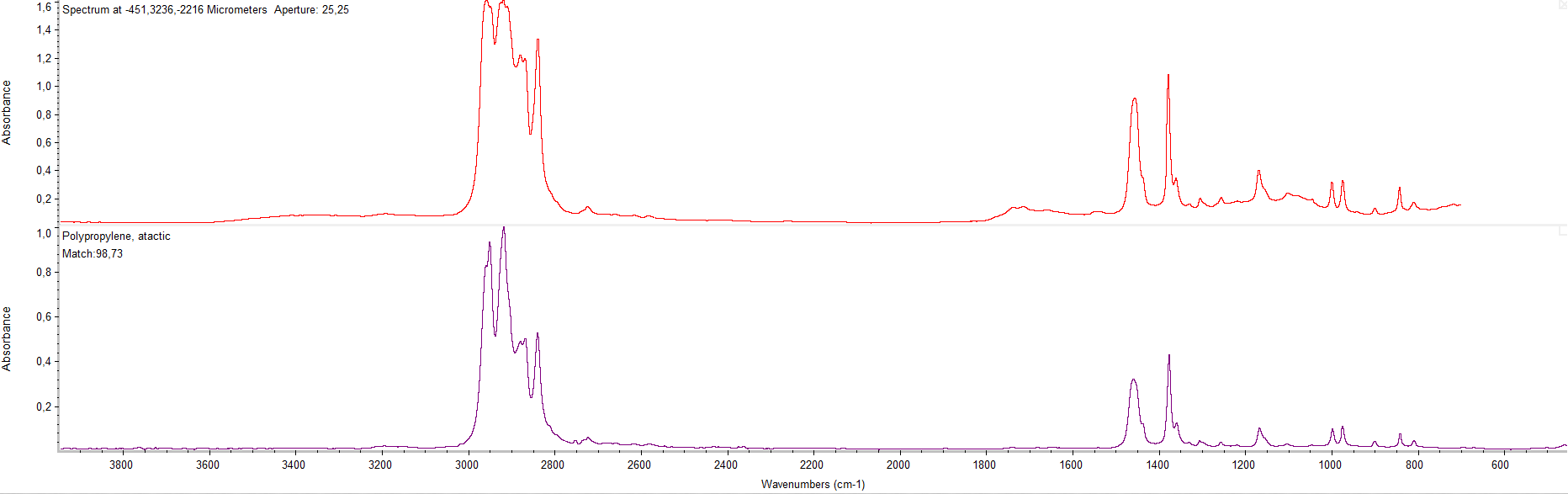


Figure S6. FTIR spectrum and spectral library search results of the small red fragment in Figure 4.


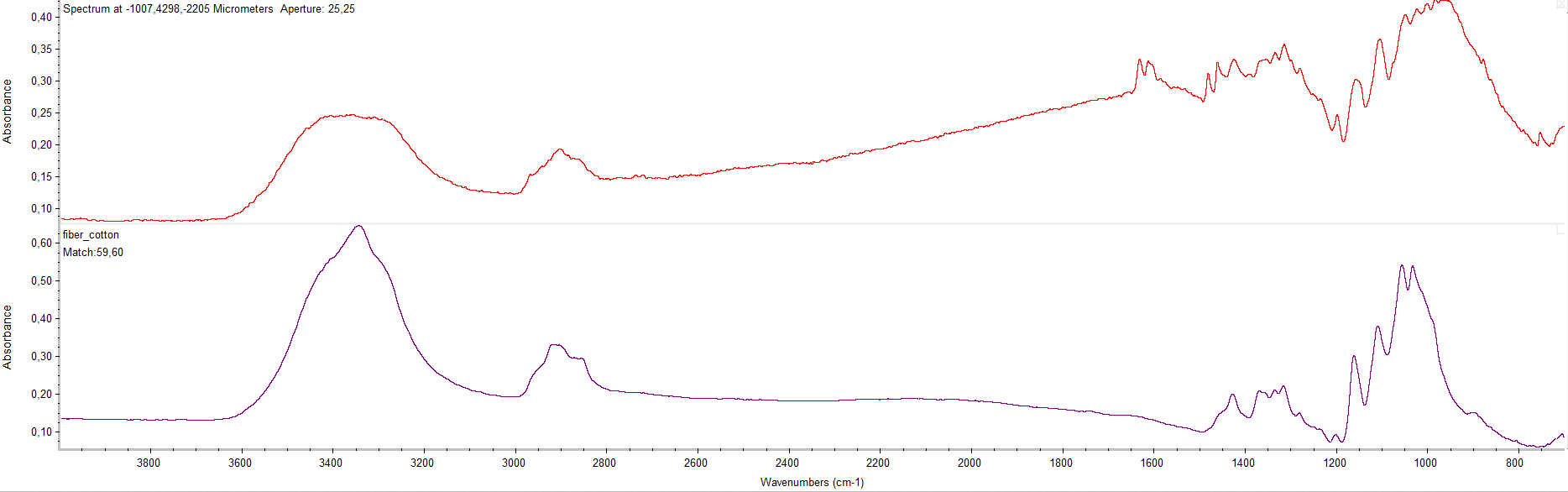


Figure S7. FTIR spectrum and spectral library search results of the small blue fiber in Figure 4. This kind of slope in the baseline is quite common in transmission FTIR analysis of microplastics, because uneven surface of particles can cause scattering effects. In this case, it lowers the match rate.


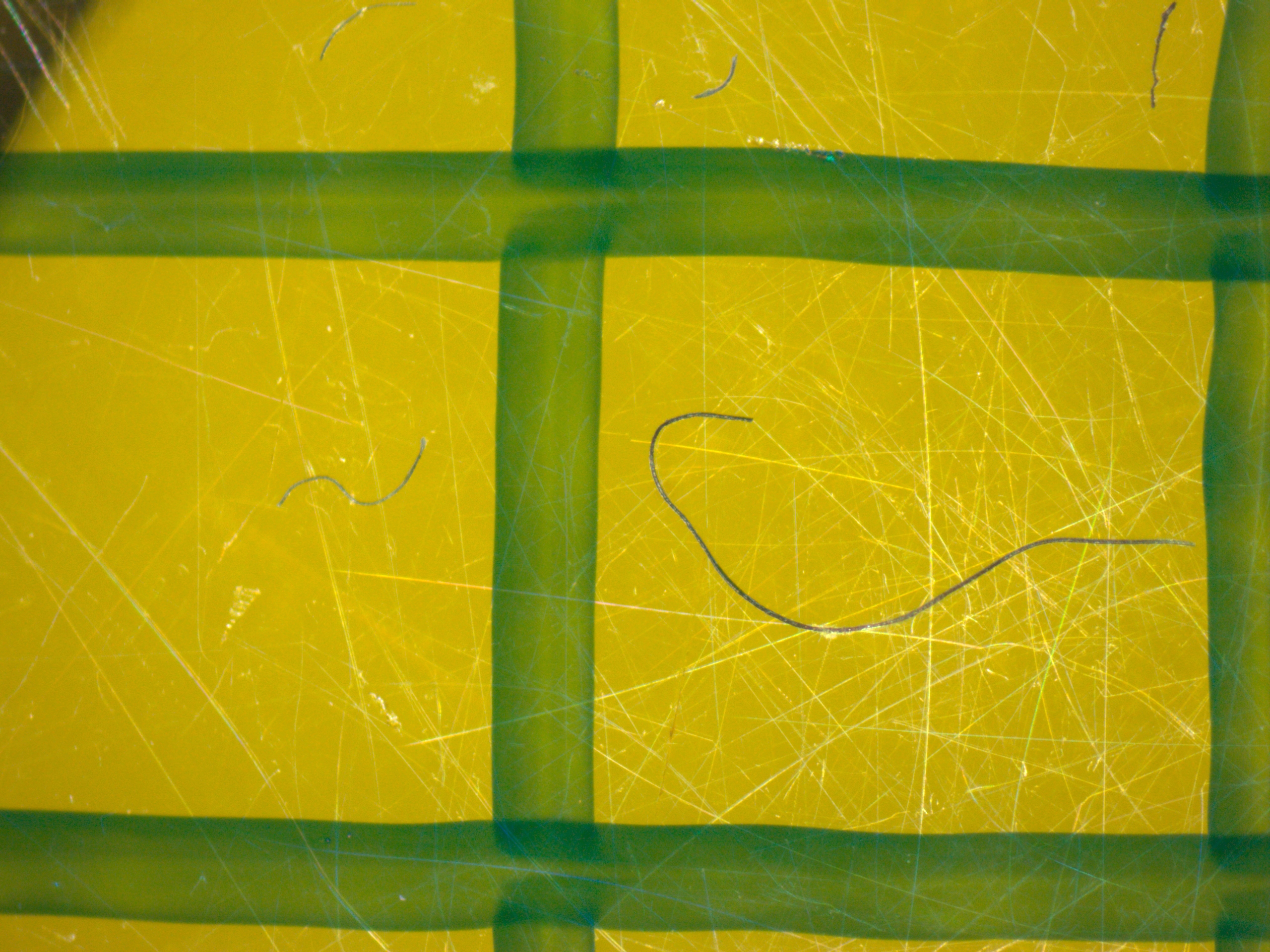


Figure S8. Particles sampled with pump filtration and 100 μm filter from site 10, WWTP. The smaller fiber in the middle is PET and the longer is PAN.


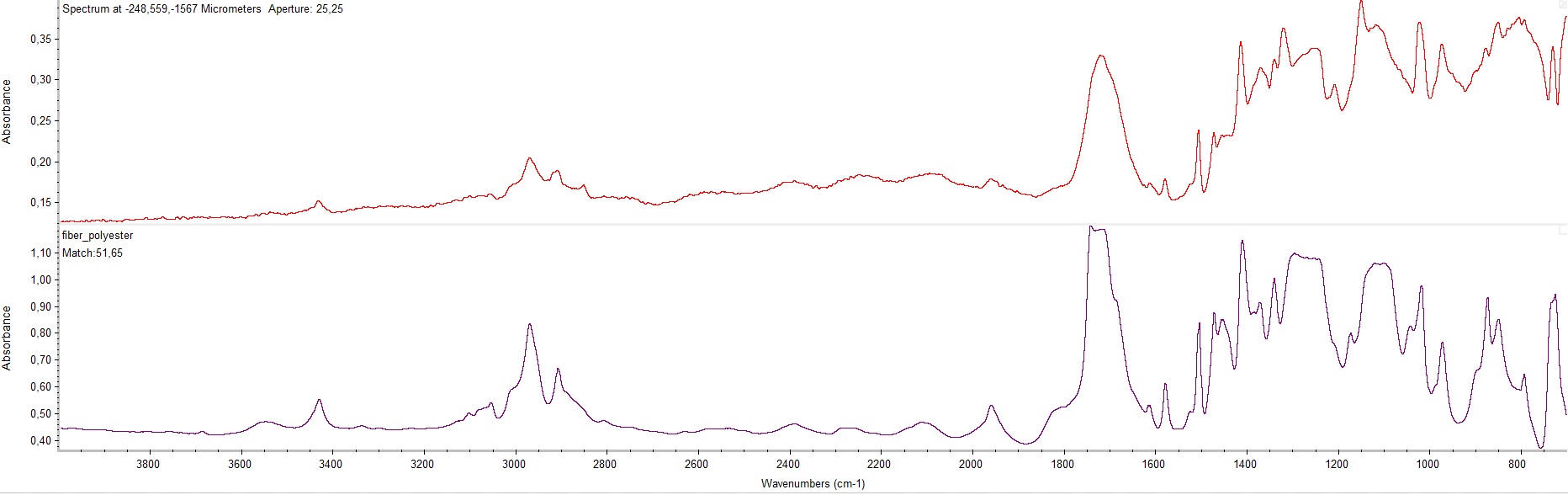


Figure S9. FTIR spectrum and spectral library search results of the shorter blue fiber in Figure 8. PET fibers were generally the most difficult plastic particle type to measure and recognize with automatic library search.


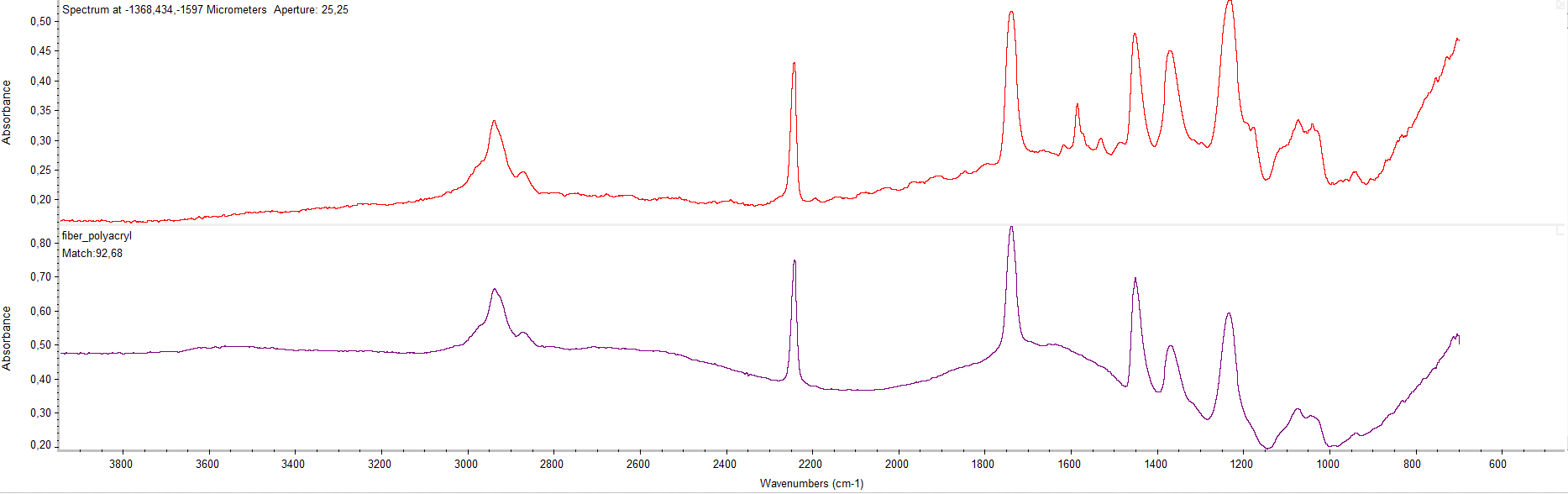


Figure S10. FTIR spectrum and spectral library search results of the longer blue fiber in Figure 8.
